# Mutational concordance between primary and metastatic melanoma: a next-generation sequencing approach

**DOI:** 10.1186/s12967-019-2039-4

**Published:** 2019-08-28

**Authors:** Antonella Manca, Panagiotis Paliogiannis, Maria Colombino, Milena Casula, Amelia Lissia, Gerardo Botti, Corrado Caracò, Paolo A. Ascierto, Maria Cristina Sini, Grazia Palomba, Marina Pisano, Melanoma Unit of Sassari (MUS), Maria Filomena Dedola, Maria Antonietta Fedeli, Maria Antonietta Montesu, Corrado Rubino, Rosanna Satta, Tiziana Scotto, Germana Sini, Italian Association for Cancer Research (AIRC) Study Group, Michele Maio, Daniela Massi, Andrea Anichini, Ulrich Pfeffer, Valentina Doneddu, Antonio Cossu, Giuseppe Palmieri, for the Italian Melanoma Intergroup (IMI), Paola Ghiorzo, Paola Queirolo, Pietro Quaglino, Vanna Chiarion Sileni, Anna Maria Di Giacomo

**Affiliations:** 1 Unit of Cancer Genetics, Institute of Biomolecular Chemistry, National Research Council, Traversa La Crucca 3, 07100 Sassari, Italy; 2 Department of Medical, Surgical, and Experimental Sciences, University of Sassari, Viale San Pietro 43, 07100 Sassari, Italy; 3 Istituto Nazionale Tumori “Fondazione Pascale”, Via Mariano Semmola, 53, 80131 Naples, Italy

**Keywords:** Skin, Cancer, Melanoma, *BRAF*, *NRAS*, Mutations, Metastasis

## Abstract

**Background:**

Cutaneous malignant melanoma (CMM) is one of the most common skin cancers worldwide. Limited information is available in the current scientific literature on the concordance of genetic alterations between primary and metastatic CMM. In the present study, we performed next-generation sequencing (NGS) analysis of the main genes participating in melanoma pathogenesis and progression, among paired primary and metastatic lesions of CMM patients, with the aim to evaluate levels of discrepancies in mutational patterns.

**Methods:**

Paraffin-embedded tumor tissues of the paired lesions were retrieved from the archives of the institutions participating in the study. NGS was performed using a specific multiple-gene panel constructed by the Italian Melanoma Intergroup (IMI) to explore the mutational status of selected regions (343 amplicons; amplicon range: 125–175 bp; coverage 100%) within the main 25 genes involved in CMM pathogenesis; sequencing was performed with the Ion Torrent PGM System.

**Results:**

A discovery cohort encompassing 30 cases, and a validation cohort including eleven Sardinian patients with tissue availability from both the primary and metachronous metastatic lesions were identified; the global number of analyzed tissue specimens was 90. A total of 829 genetic non-synonymous variants were detected: 101 (12.2%) were pathogenic/likely pathogenic, 131 (15.8%) were benign/likely benign, and the remaining 597 (72%) were uncertain/unknown significance variants. Considering the global cohort, the consistency in pathogenic/pathogenic like mutations was 76%. Consistency for *BRAF* and *NRAS* mutations was 95.2% and 85.7% respectively, without statistically significant differences between the discovery and validation cohort.

**Conclusions:**

Our study showed a high level of concordance in mutational patterns between primary and metastatic CMM, especially when pathogenic mutations in driver genes were considered.

## Background

Cutaneous malignant melanoma (CMM) is one of the most common skin cancers worldwide [1]. CMM incidence constantly increases in the last decades, and mortality rates rise, especially in white males [2]. CMM mortality is higher in advanced stage cases, not suitable for complete surgical removal, in which traditional chemotherapy is often characterized by poor oncological benefits [3]. Consistent improvements in survival in this subset of patients were obtained with the introduction of targeted and immunological therapies in recent years. Actually, targeted therapies are performed with the combination of BRAF inhibitors (dabrafenib, vemurafenib, encorafenib) and MEK inhibitors (cobimetinib, trametinib, binimetinib) in patients with CMM carrying a *BRAF* mutation (approximately, 50% of the cases), MEK inhibitors alone in *BRAF* wild-type cases with *NRAS* mutations, and KIT inhibitors (imatinib, nilotinib, etc.) in patients with *KIT* mutated lesions [4]. In other words, targeted therapies are based on the knowledge of specific genetic alterations occurring in the tumors to treat. Unfortunately, tumors are not static, but dynamic entities and their mutational landscape continuously changes during their progression from premalignant lesions to metastasis, and can also be influenced by the therapeutic interventions adopted.

Several studies have been performed in the past to evaluate the concordance of *BRAF* and *NRAS* mutations between primary tumors and their metastases in order to better understand the pathophysiology of the metastatic process in CMM, and to respond on specific clinical issues regarding the quality of mutational analysis is tissue from metastases in comparison to that performed in the primary tumor [5]. Some of these studies showed a good concordance between primary tumors and lymph node or visceral metastases, but low rates when soft tissue metastatic samples were compared with the primary lesions [6–8]. Nevertheless, studies with consistently lower concordance rates, also between primary and lymph node or visceral metastases, have been published [9, 10]. Most of these reports regarding *BRAF* and *NRAS* used a single conventional method for identifying the mutations, and enrolled small cohorts, undermining the validity of conclusions, and making further investigations necessary. Additional studies employing conventional sequencing evaluated the concordance of the specific mutations on other genes participating in the oncogenic process of CMM, like *CDKN2A*, *MITF*, *EGFR*, *CCND1*, *cMET*, and *cKIT* and others, evidencing differences in genes selected during tumor progression (like *CDKN2A*, *MITF*, etc.) [11]. Globally, all the studies mentioned elucidated only a small frame of the global change of the mutational landscape of metastatic CMM, in comparison to the origin tumors.

The advent of next-generation sequencing (NGS) technologies for genetic testing accelerated the efforts to identify the whole pattern of mutations involved in the CMM pathogenesis [12]. Recent whole exome (WES) or genome sequencing (WGS) studies provided precious details regarding genetic alterations in numerous genes included in a wide range of molecular pathways and networks in melanomagenesis and thus allowing the molecular sub-classification of the several types of melanoma [13–17]. Nevertheless, limited information is available on the concordance of the genetic alterations reported between primary and metastatic melanomas; such information is crucial for the comprehension of the single roles and the interplay between specific genetic events in the metastatic process, as well as for establishing the validity of testing in CMM metastatic tissue for all the mutations in genes used in clinical practice for current and future targeted therapies. In the present study, we performed NGS-based analysis of the main genes participating in melanoma pathogenesis and progression, included in a specific gene-panel designed by the Italian Melanoma Intergroup (IMI) on the basis of previous studies, in paired primary and metastatic lesions of patients with CMM, with the aim to evaluate potential discrepancies in mutational patterns. Although a more detailed picture of the pathogenic changes could be obtained with larger gene panels or, more extensively, WES/WGS screening approaches, the use of a panel containing a limited number of driver genes may be much easier to be introduced into the clinical practice.

## Materials and methods

### Patients

Consecutive Italian patients with a histologically proven diagnosis of metastatic CMM from January 2009 to December 2017 were retrieved from the archives of the southern Italy anatomic pathology institutes participating in the study, and cases with tissue availability from both the primary and at least one metachronous lymph node or visceral metastatic lesion were identified. Metastatic melanomas were considered as metachronous when melanoma metastasis was diagnosed after at least 6 months from the diagnosis of the primary melanoma. Patients with soft tissue metastases were excluded because of the low concordance in mutational rates in comparison with the primary lesions described in older studies, as mentioned above [6–8]. Brain metastases samples were not available in any case. In addition, using the same criteria, a validation cohort of consecutive Sardinian patients with available paired primary and metastatic CMM samples was identified from the archives of the Anatomic Pathology Unit of the University of Sassari, within the same time frame. The demographic, clinical and pathological data of all patients were retrieved from clinical records and reports. All the patients gave their informed consent for the use of their clinical data for the purposes of the study. The study was performed in accordance with the principles of the declaration of Helsinki and was approved by the Committee for the Ethics of the Research and Bioethics of the National Research Council (CNR).

### Molecular analysis

**Fig. 1:**
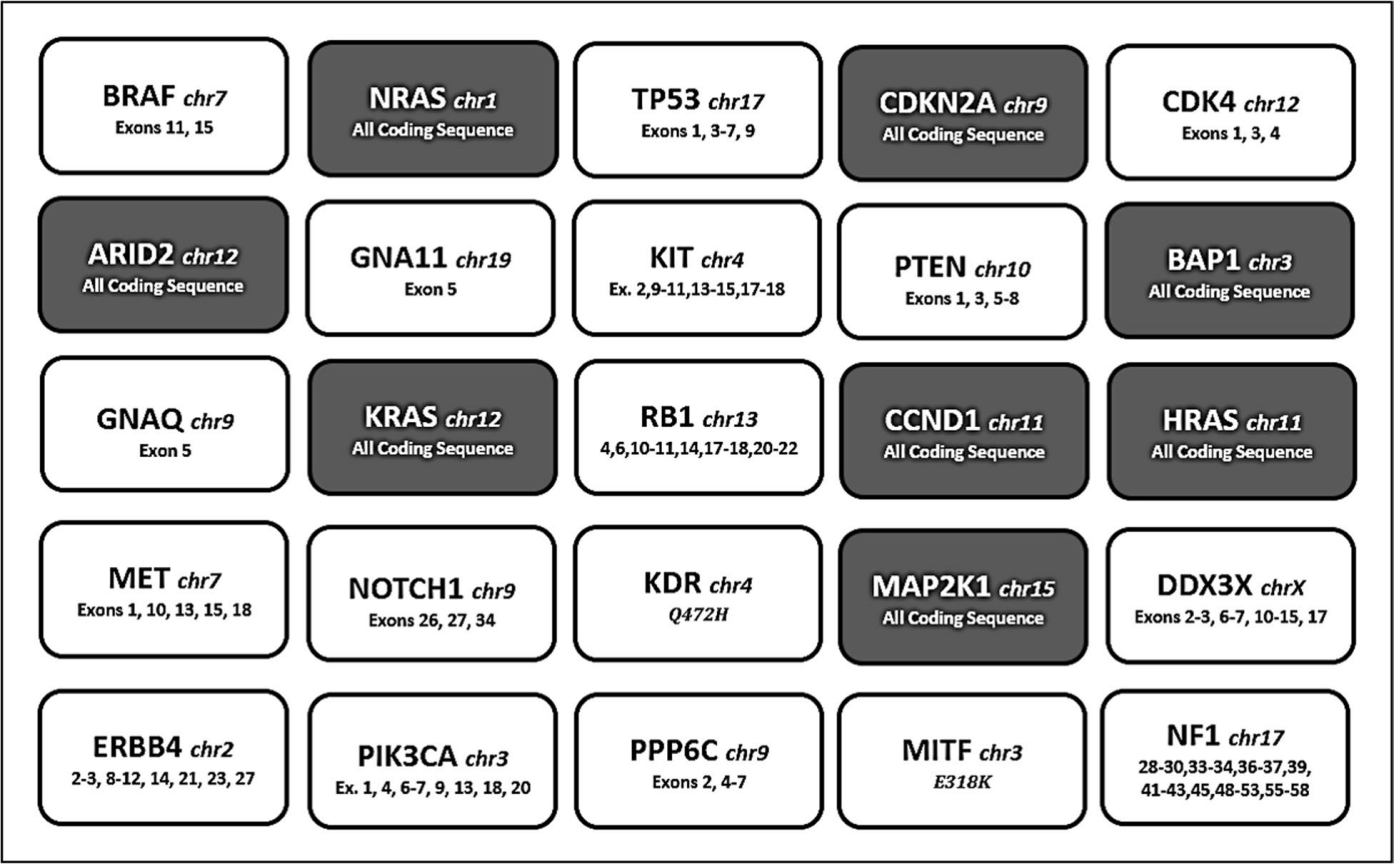
The Italian Melanoma Intergroup (IMI Somatic DNA panel) used for genetic testing including 343 amplicons, size range 125–175 bp, coverage 100%, within the main 25 genes involved in the pathogenesis of melanoma

For mutation analysis, paraffin-embedded tumor tissues of the paired lesions were retrieved from the pathological archives of the institutions participating in the study. Using light microscopy, tissue sections were selected in order to obtain tumor samples with at least 80% neoplastic cells. Genomic DNA was isolated using the GeneRead DNA FFPE Kit (Qiagen, Hilden, Germany), following the manufacturer´s instructions. NGS was performed using a specific multiple-gene panel constructed by the IMI (IMI somatic DNA panel), arranged in three primer pools, and designed using the Ion AmpliSeq Designer to explore the mutational status of selected regions (343 amplicons; amplicon range: 125–175 bp; coverage 100%) within the 25 genes reported as the most frequently mutated in CMM specimens by The Cancer Genome Atlas (TGCA) and successive NGS-based studies [12, 13]. Figure 1 summarizes the characteristics of the IMI panel. Although this is the first time the IMI gene panel is used in a study, several genomic DNA samples from FFPE melanoma tissues were blindly analyzed in separate Italian laboratories using different NGS platforms in order to achieve full validation of the IMI panel for mutation pattern detection at somatic level (Ghiorzo and Palmieri, manuscript in preparation). Barcoded amplicon libraries were generated from 10 ng template DNA × primer pool and purified with AMPure beads (Beckman Coulter, Brea, CA, USA). Purified DNA was diluted at a final concentration of 50 pM, placed into the Ion Chef for emulsion PCR and Chip (316 v2BC) loading, and sequenced on the Ion Torrent PGM System (Life Technologies, Waltham, MA, USA). Sequencing data were processed with the Ion Torrent platform-specific pipeline software (Torrent Suite, V5.2.1; Life Technologies); the Ion Reporter and Integrative Genome Viewer were used for variant annotation and reads visualizations, respectively (http://www.broadinstitute.org/igv).

Coverage of > 300 reads and frequency of mutated alleles > 3% for gene amplicon, in order to get a total amount of at least 10 mutated alleles for each candidate amplicon, were adopted for mutation selection criteria at somatic level. In the discovery cohort, a total of 844,153 reads was achieved for selecting 750 nucleotide variants, with an average of 1125 reads per mutated gene amplicon (range, 302 to 2000). In the validation cohort, a total of 70,576 reads was achieved for selecting 79 nucleotide variants, with an average of 893 reads per mutated gene amplicon (range, 302 to 2000). Sequence variants were classified as pathogenic, likely pathogenic, uncertain significance, likely benign, or benign, according to their capability to either affect the function of the gene or be plausibly linked to the disease. In particular, pathogenicity was assessed through data comparisons using the following sequence databases: the ClinVar archive of reports of relationships among medically relevant variants and phenotypes (http://www.ncbi.nlm.nih.gov/clinvar/) and the Catalogue Of Somatic Mutations In Cancer (COSMIC v88; https://cancer.sanger.ac.uk/cosmic).

All mutations in melanoma driver oncogenes (*BRAF* and *NRAS*) and a fraction of randomly-selected pathogenic variants with high rates of the mutated alleles in the remaining genes were confirmed by Sanger sequencing of gene-specific amplicons. Briefly, polymerase chain reaction (PCR) was performed on 20 ng of genomic DNA in a Veriti 96-Well Fast Thermal Cycler (Life Technologies-ThermoFisher Scientific); all PCR-amplified products were directly sequenced using an automated fluorescence-cycle sequencer (ABI3130, Life Technologies). Sequencing analysis was conducted in duplicate and in both directions (forward and reverse) for all evaluated samples.

### Statistical analysis

Results were expressed as percentages, mean (mean ± SD) or median values (median and IQR). Variables distribution was assessed by the Shapiro–Wilk test. Statistical differences were assessed using the unpaired Student’s t-test or Mann–Whitney rank sum test, the Chi-square test or Fisher’s exact test as appropriate. Correlations between clinical and genetic variables were assessed by Pearson’s or Spearman’s correlation, as appropriate. Statistical analyses were performed using MedCalc for Windows, version 15.4 64 bit (MedCalc Software, Ostend, Belgium).

## Results

**Fig. 2:**
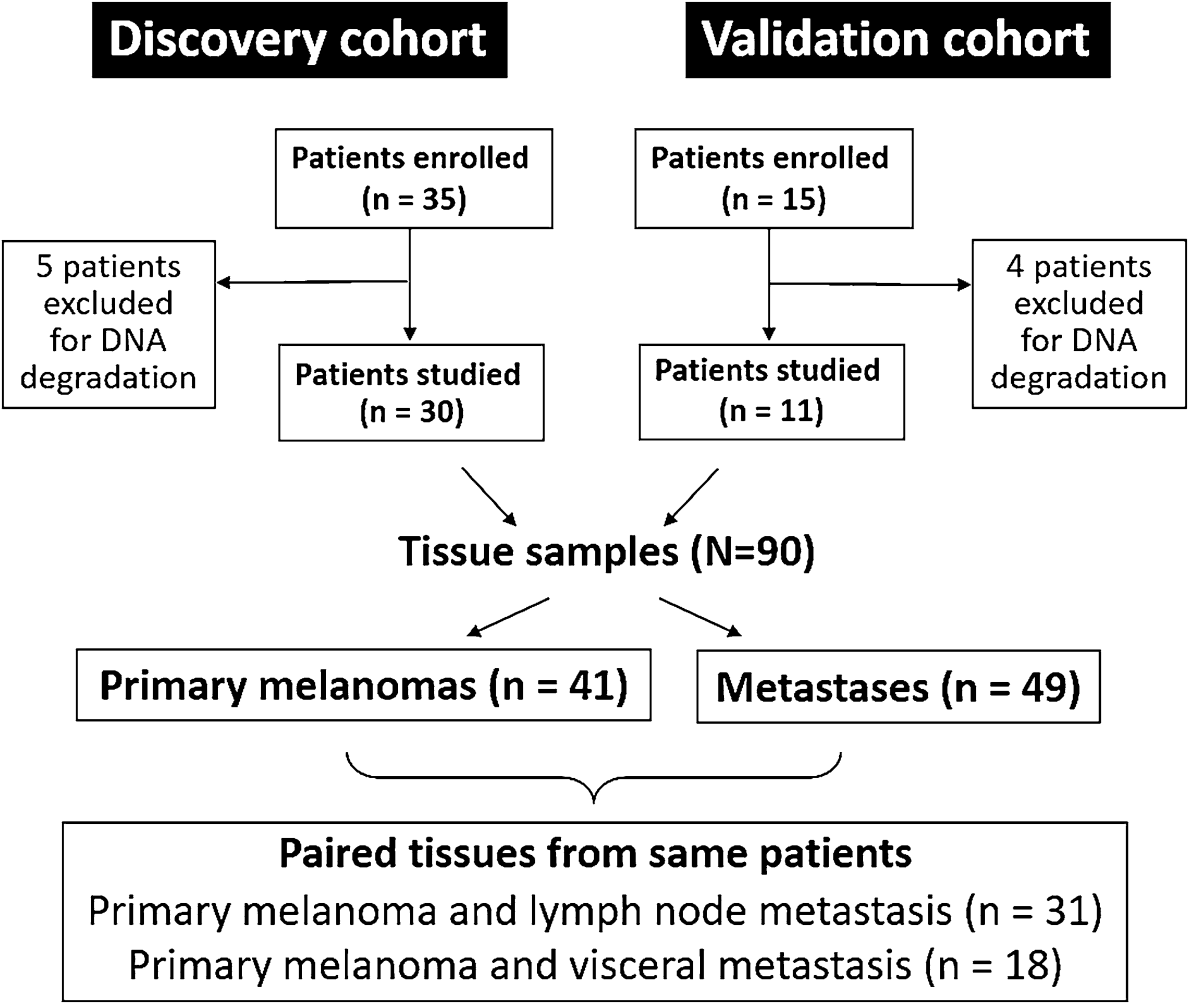
Description of the cohorts enrolled in the study

Thirty-five national cases with tissue availability from both the primary and at least one metachronous lymph node or visceral metastatic lesion were identified. Among them, five patients were excluded because of the low quality of the DNA extracted, and thus, the remaining 30 were enrolled in the study. Among the 15 Sardinian cases identified, eleven patients were enrolled and four were excluded because of sample DNA degradation. The global number of patients enrolled was 41, and the total of tissue samples retrieved 90 (Fig. 2); the primary CMMs were 41 and the metastatic lesions 49. Paired primary and lymph node metastasis specimens were available in 31 cases, while paired primary and visceral metastasis samples were obtained in 18 cases (nine liver, eight lung, and one small intestine metastasis).

The main demographic, clinical and pathological characteristics of the cohorts included in the study are summarized in Table 1; no statistically significant differences were found in such characteristics between the discovery and validations groups, with the exception of sex (all patients in the validation cohort were males) and the number of mitoses in the primary lesions which were significantly lower in the validation group.

**Table 1:** Demographic, clinical and pathological features of the patients included in the study

Characteristics	Global cohort (41 cases)	Discovery cohort (30 cases)	Validation cohort (11 cases)	p-value
Male sex, n (%)	28 (68.3)	15 (53.6)	11 (100)	*0.003*
Age, (mean ± SD), years	55 ± 12.7	53.9 ± 13.2	58 ± 11.1	0.324
IPMD, (mean ± SD), months	24.3 ± 26.4	25.3 ± 29.2	21.1 ± 13.4	0.523
Melanoma type, n (%)				
NM	12 (29.3)	8 (26.7)	4 (36.4)	0.828
SSM	28 (68.3)	21 (70)	7 (63.6)	0.993
LMM	1 (2.4)	1 (3.3)	0 (0)	1.000
Melanoma site, n (%)				
A. Primitive				
Head	2 (4.9)	2 (6.7)	0 (0)	1.000
Neck	3 (7.3)	2 (6.7)	1 (9.1)	1.000
Trunk	19 (46.3)	13 (43.3)	6 (54.5)	0.776
Upper limbs	3 (7.3)	2 (6.7)	1 (9.1)	1.000
Lower limbs	14 (34.1)	11 (36.7)	3 (27.3)	1.000
B. Metastasis	49 (100)	36 (73.5)	13 (26.5)	0.853
Lymph nodes	31 (64.6)	23 (63.9)	8 (66.7)	
Visceral	18 (35.4)	13 (36.1)	5 (33.3)	
Number of mitosis per smm (mean ± SD)	3.4 ± 3.1	4.1 ± 3.0	1.6 ± 2.5	*0.003*
Breslow thickness, (mean ± SD)	3.8 ± 2.4	4.0 ± 2.4	3.2 ± 1.4	0.566
Ulceration, n (%)	18 (43.9)	12 (40)	6 (54.5)	0.634
Initial T/N stage, n (%)				
A. T stage				
T1	2 (4.9)	2 (6.7)	0	1.000
T2	7 (17.1)	5 (16.7)	2 (18.2)	1.000
T3	19 (46.3)	13 (43.3)	6 (54.5)	0.776
T4	13 (31.7)	10 (33.3)	3 (27.3)	1.000
B. N stage				
N0	8 (19.5)	6 (20.0)	2 (18.2)	1.000
N1	16 (39.0)	11 (36.7)	5 (45.4)	0.723
N2	13 (31.7)	9 (30.0)	4 (36.4)	1.000
N3	4 (9.8)	4 (13.3)	0	0.559

Significant p-values are indicated in italics

*IPMD* interval progression of metastatic disease, *LMM* lentigo maligna melanoma, *NM* nodular melanoma, *SD* standard deviation, *SSM* superficial spreading melanoma

A total of 829 genetic variants were detected in all the 90 lesions examined; the incidence of the variants was significantly higher in the discovery cohort (750 variants) in comparison to the validation cohort (79 variants, p = 0.001). All the genetic variants detected are included in Additional file 1: Table S1. The variants were classified as pathogenic/likely pathogenic, benign/likely benign, and uncertain/unknown significance variants in accordance with the COSMIC and ClinVar databases as mentioned above (Additional file 2: Table S2); globally, 101 (12.2%) variants were pathogenic/likely pathogenic, 131 (15.8%) were benign/likely benign, and the remaining 597 (72%) were uncertain/unknown significance variants. The pathogenic/likely pathogenic variants affected with higher frequency the discovery than the validation cohort (87 vs. 14), but the difference was not statistically significant (p = 0.117). Furthermore, these variants were equally distributed between primary and metastatic tumors (49 vs. 52). Half of the pathogenic/likely pathogenic variants involved the *BRAF* gene (50 variants, 49.5%); other genes harboring such variants were *NRAS* (14 variants, 13.9%), *TP53* (14 variants, 13.9%), *CDKN2A* (4 variants, 4%), and others (Additional file 2: Table S2). Conversely, benign/likely benign variants were most often harbored in the *TP53* (45 variants, 34.3%), *KDR* (24 variants, 18.3%), *PIK3CA* (21 variants, 16%), and *KIT* (20 variants, 15.3%) genes.

Considering the global cohort, the consistency in pathogenic/pathogenic like mutational patterns between primary and metastatic melanomas was 76% (Table 2). Consistency was higher between primary lesions and lymph node metastasis than between primary tumors and visceral metastasis, with the difference being statistically significant (p = 0.019, Table 2). Furthermore, global primary tumor-metastasis consistency was slightly higher in the validation cohort, than in the discovery cohort, but the difference was not statistically significant (p = 0.708). Concordance was slightly reduced (63%) when the functionally known variants (pathogenic/likely pathogenic + benign/likely benign) were considered together in the whole cohort, and was significantly lower when all the variants were pooled together (24%, p = 0.001) (Table 3). We also searched for statistically significant differences in pathogenic/likely pathogenic mutations concordance by sex, age, and time to metastasis. No sex predilection was found comparing discordant with concordant cases (p = 0.722), as well as no statistical differences in age [52.5 (IQR 50–67.5) vs. 53 (IQR 46.2–61.5) years, p = 0.504] and the time to metastasis [13.5 (IQR 11–40) vs. 18 (0–25.7) months, p = 0.297]. Similar results were obtained dividing the patients in age groups (less than 50 years vs. 50 years or more, p = 0.466), and in groups by the time to metastasis (shorter vs. longer than 24 months, which was the mean time to metastasis observed, p = 0.726).

**Table 2:** Consistency between pathogenic/likely pathogenic mutation patterns in paired primary and metastatic lesions: A—all cases, B—discovery cohort, C—validation cohort

Paired tissue types	No. of samples	Cases with consistent mutation pattern (%)	Cases with discrepant mutation pattern (%)
A. All cases			
Primary vs. lymph node metastasis	31	27 (87)	4 (13)
Primary vs. visceral metastasis	18	10 (56)	8 (44)
Primary vs. metastasis	49	37 (76)	12 (24)
B. Discovery cohort			
Primary vs. lymph node metastasis	23	20 (87)	3 (13)
Primary vs. visceral metastasis	15	8 (53)	7 (47)
Primary vs. metastasis	38	28 (74)	10 (26)
C. Validation cohort			
Primary vs. lymph node metastasis	8	7 (87.5)	1 (12.5)
Primary vs. visceral metastasis	3	2 (67)	1 (33)
Primary vs. metastasis	11	9 (82)	2 (18)

**Table 3:** Consistency between variant patterns in paired primary and metastatic lesions: A—classified (pathogenic/likely pathogenic and benign/likely benign) variants, B—all variants

Paired tissue types	No. of samples	Cases with consistent mutation pattern (%)	Cases with discrepant mutation pattern (%)
A. Pathogenic/likely pathogenic + benign/likely benign variants			
Primary vs. lymph node metastasis	31	23 (74)	8 (26)
Primary vs. visceral metastasis	18	8 (44)	10 (56)
Primary vs. metastasis	49	31 (63)	18 (37)
B. All variants			
Primary vs. lymph node metastasis	31	9 (29)	22 (71)
Primary vs. visceral metastasis	18	3 (17)	15 (83)
Primary vs. metastasis	49	12 (24)	37 (76)

We also evaluated the concordance of pathogenic/likely pathogenic mutations in the main single genes involved in clinical practice for the prescription of targeted therapies (Table 4). Consistency for *BRAF* mutations was 95.2% (V600E, V600K, and G469S variants) present in both the primary and metastatic tumors. Similarly, consistency for *NRAS* mutations was 85.7%; one patient had an *NRAS* mutation in the primary tumor and no mutation in the corresponding visceral metastasis examined. No significant differences were observed between the discovery and the validation cohort.

**Table 4:** Consistency in *BRAF* and *NRAS* pathogenic/likely pathogenic variants in our cohort

	Cases with a mutation in the primary tumor	Consistent mutation pattern with metastasis (%)
*BRAF* mutations		
Primary vs. lymph node metastasis	18	17 (94.4)
Primary vs. visceral metastasis	3	3 (100)
Primary vs. metastasis	21	20 (95.2)
Discovery cohort	17	17 (100)
Validation cohort	4	3 (75)
*NRAS* mutations		
Primary vs. lymph node metastasis	3	3 (100)
Primary vs. visceral metastasis	4	3 (75)
Primary vs. metastasis	7	6 (85.7)
Discovery cohort	5	4 (80)
Validation cohort	2	2 (100)

## Discussion

Cutaneous malignant melanoma, like most human cancers, is a disease resulting from a dynamic pathogenic process characterized by the accumulation of genetic alterations in the neoplastic cells, under the pressure of several oncogenic stimuli. The main genetic alterations necessary for melanomagenesis and early progression, have been widely elucidated [18]; less is known about the genetic mutational patterns determining and characterizing regional and distant melanoma metastasis. The latter issue is particularly interesting, also for practical reasons, in order to determine the clinical validity of mutational testing performed in metastatic biopsy specimens.

What can undermine the validity of these tests is the occurrence of intratumoral and intertumoral heterogeneity. A high number of clones harboring various mutations contribute to a great level of intratumor heterogeneity of CMM and generate metastases which may originate from different subclones. Multiple molecular events on a genomic (point mutations, deletions, aberrations, etc.), transcriptomic/proteomic (over-, under-expression of genes, etc.), and epigenetic (methylation, micro-RNA and long non-coding RNA regulation, etc.) level can additionally contribute in further increase such heterogeneity [19]. Indeed, all these levels contributed to the molecular heterogeneity evidenced in The Cancer Genome Atlas (TCGA) study, in which across the eleven different cancer types included, there were 4473 primary tumor samples (104 from melanoma) and 395 tumor metastasis samples (including 369 from melanoma), but only 29 paired cases from the same patient, and external to the TCGA datasets were analyzed [20]. Moreover, the introduction of newly conceived targeted therapies has been demonstrated able to impact the mutational landscape of melanomas, creating further pressure on clonality, and molecular alterations at all the levels mentioned [21]. This influences, not only the validity of the diagnostic tests but also the effectiveness of the therapeutic strategies adopted and therefore dictates a better knowledge of the variations occurring during the course of the disease.

The incidence of the main pathogenic mutations displaying critical roles in melanomagenesis (*BRAF*: 49.5%, *NRAS*: 13.9%, *TP53*: 13.9%) in our study was similar to that published in previous studies performed with NGS techniques, with the exception of *KIT* mutations. De Unamuno Bustos et al., Reiman et al., and Siroy et al. sequenced samples from 100, 151 and 699 CMM cases, with custom Ampliseq panels or pan-cancer hot spot NGS panels [22–24]. In these studies, the frequency of *BRAF*, *NRAS*, and *KIT* mutations was respectively 36–50%, 15–27%, 4–5%; in our study no pathogenic *KIT* variants were detected, while several unknown/uncertain and benign/likely benign were encountered. Similarly, in a previous study performed by the IMI using the AmpliSeq Cancer Panel HotSpot V2/CHPv2 on the Ion Torrent platform which investigates approximately 2800 mutations in 50 most common oncogenes and tumor suppressor genes, only *KIT* polymorphisms, but no mutations, were detected [25]. In a further study performed with conventional methods in the Italian population, including Sardinian patients, *KIT* amplifications were detected in 3.3% of the primary and 5.4% of the metastases examined [11].

Previous studies reported that the number of mutations in genes involved in the MAPK pathway, including *BRAF* and *NRAS*, was increased from premalignant lesions to melanoma; it was therefore stated that MAPK becomes activated at the earliest stage of neoplasia and progressively ramps up as malignant transformation proceeds [13, 26]. Nevertheless, this process seems to be completed in the early phases of melanomagenesis, because MAPK pathway mutations are constantly present in metastatic tissues with similar percentages as in primary lesions, with *BRAF* and *NRAS* mutations as almost mutually exclusive genetic events [27]. Shein et al. examined 12 pairs of primary CMM and the corresponding regional metastases and found most of the pathogenic mutations were shared between primary and metastatic lesions; other additional private mutations were detected, as occurred in our cohorts, but there is no evidence that their selection was associated with the metastatic spread [26]. In the study of Miraflor et al. performed with an NGS panel consisting of 207 amplicons covering over 20,000 bases across 50 genes with known cancer associations, a total of 8 patients with paired specimens were screened for somatic mutations [28]. Among them, four cases showed the same mutations in their metastatic lesions from different sites (*ATM, NRAS, TP53, BRAF* and *JAK3* mutations), while the remaining four patients harbored different gene mutations at metastatic sites compared to their primary lesions or metastasis from different sites (*BRAF, CDKN2A, PIK3CA*, and *ATM* mutations).

In our previous study, performed with the AmpliSeq HotSpot cancer panel, asynchronous (9 cases) and synchronous (16 cases) metastatic lymph nodes and the corresponding primary melanoma tissues were sequenced and no significant differences in *BRAF/NRAS* mutation rates between primary (19 of 25; 76%) and metastatic (39 of 50; 78%) lesions were observed, indicating that *BRAF/NRAS* mutations may occur early in melanoma development, and their incidence may remain quite unvaried during melanoma progression [25].

Our current results confirmed the high consistency level of pathogenic/likely pathogenic mutations between the primary tumors and the lymph node metastasis (87%). Concordance rates, were significantly lower when the visceral lesions were tested in comparison to lymph node metastases. This could raise some concerns, as current clinical guidelines recommend to perform mutational analysis on metastatic tissue in patients with advanced stage CMM, and if unavailable, to test the primary lesions [29, 30]. Nevertheless, when the pathogenic mutations to compare were restricted to the BRAF and NRAS activating variants, the concordance was higher irrespective of the metastatic site, confirming that genetic analysis can be performed in both types of lesions. The decreasing trend of consistency in pathogenic variants from primary to regional and then to distant metastasis supports the theory of the accumulation of genetic alterations during the linear progression of CMM, on which is based the surgical removal of lymph nodes with curative intent. Concordance rates do not seem to be influenced by sex, age or time to metastasis. The higher mutational discrepancies were observed in previous studies in soft tissue and brain metastases [6, 31], and for this reason, we decided to exclude these subsets of patients, which need specific studies and alternative guideline recommendations.

In our study, and in most of the previous studies mentioned, high rates of concurrent *BRAF* (55%) and *NRAS* (20%) mutations were detected [26]. Furthermore, a great number of uncertain/unknown genetic variants was found. It is hard to predict the pathophysiological and clinical impact of these variants, and if they are or not passenger alterations which sporadically influence specific phases of the metastatic process. The validation cohort in our study was from Sardinia and had globally a lower incidence of these variants; this may be dependent on the genetic peculiarity of island populations. In Sardinia, whose population shows a high level of genetic homogeneity due to geographical isolation and strong genetic drift, different mutation rates in several driver oncogenes were already demonstrated for various types of cancer by our group [32, 33], strongly suggesting that different “genetic background” may also induce discrepant penetrance and distribution of somatic mutations in candidate cancer genes. Overall, most of these genetic variants do not display relevant roles in the metastatic process, as their absence does not prevent or attenuate it.

Our study has some limitations, mainly the low number of cases, the retrospective approach used in selecting them, and the lack of data regarding the therapies employed for the clinical management of the patients during the evolution of the disease. We are aware that a larger collection of CMM patients with highly detailed clinical information could permit to also make comparisons between the concordant or discrepant alterations in driver genes and additional factors involved into the disease behavior (i.e. responsiveness or resistance to therapies, immune status, etc.). On the other hand, this is the first specifically designed study to investigate a tailored CMM panel of genetic alterations in primary and lymph node and visceral metastatic lesions, with an NGS approach, and in any case, includes the higher number of paired primary—metastatic tissues evaluated this way.

## Conclusions

Our research showed a high level of concordance in mutational patterns between primary and metastatic CMM. Consistency was higher for pathogenic/likely pathogenic variants, which involved mainly the *BRAF*, *NRAS* and *TP53* genes. Furthermore, consistency was higher between primary tumors and the corresponding lymph node metastasis, rather than visceral metastasis. Nevertheless, consistency for the main genes implicated in clinical practice (*BRAF and NRAS*) was extremely high, confirming previous evidence suggesting that metastatic or primary tissue can both be effectively used for mutational analysis. A high number of unknown/uncertain variants were detected in both primary and metastatic lesions, and their role remains to be elucidated in future studies (Additional file 3: Table S3).

## Supplementary information

**Additional file 1: Table S1.** (A) The 750 somatic non-synonymous variants found in discovery cohort, in detail. In bold, variants classified as pathogenic/likely pathogenic mutations. (B) The 79 somatic non-synonymous variants found in validation cohort, in detail. In bold, variants classified as pathogenic/likely pathogenic mutations.

**Additional file 2: Table S2.** Gene variants in paired melanoma samples. Asterisks indicate different variant types within the same patient.

**Additional file 3: Table S3.** Gene variant patterns in paired melanoma samples. In gray, cases withconcordant patterns for pathogenic/likely pathogenic variants.

## Data Availability

The datasets used and/or analysed during the current study are available from the corresponding author on reasonable request.

## Abbreviations

CMM: cutaneous malignant melanoma
CNR: National Research Council
IMI: Italian Melanoma Intergroup
IQR: interquartile range
NGS: next-generation sequencing
PCR: polymerase chain reaction
SD: standard deviation
TGCA: The Cancer Genome Atlas
WES: whole exome sequencing
WGS: whole genome sequencing
